# Structure of the Hydrophobic Core Determines the 3D Protein Structure—Verification by Single Mutation Proteins

**DOI:** 10.3390/biom10050767

**Published:** 2020-05-14

**Authors:** Mateusz Banach, Piotr Fabian, Katarzyna Stapor, Leszek Konieczny, Irena Roterman

**Affiliations:** 1 Department of Bioinformatics and Telemedicine, Medical College, Jagiellonian University, Lazarza 16, 31-533 Krakow, Poland; mateusz.banach@uj.edu.pl; 2 Institute of Computer Science, Silesian University of Technology, Akademicka 16, 44-100 Gliwice, Poland; piotr.fabian@polsl.pl (P.F.); katarzyna.stapor@polsl.pl (K.S.); 3 Chair of Medical Biochemistry, Medical College, Jagiellonian University, Kopernika 7, 31-034 Krakow, Poland; mbkoniec@cyf-kr.edu.pl

**Keywords:** hydrophobicity, hydrophobic core, secondary structure, super-secondary structure, synergy

## Abstract

Four de novo proteins differing in single mutation positions, with a chain length of 56 amino acids, represent diverse 3D structures: monomeric 3α and 4β + α folds. The reason for this diversity is seen in the different structure of the hydrophobic core as a result of synergy leading to the generation of a system in which the polypeptide chain as a whole participates. On the basis of the fuzzy oil drop model, where the structure of the hydrophobic core is expressed by means of the hydrophobic distribution function in the form of a 3D Gaussian distribution, it has been shown that the composition of the hydrophobic core in these two structural forms is different. In addition, the use of a model to determine the structure of the early intermediate in the folding process allows to indicate differences in the polypeptide chain geometry, which, combined with the construction of a common hydrophobic nucleus as an effect of specific synergy, may indicate the reason for the diversity of the folding process of the polypeptide chain. The results indicate the need to take into account the presence of an external force field originating from the water environment and that its active impact on the formation of a hydrophobic core whose participation in the stabilization of the tertiary structure is fundamental.

## 1. Introduction

The three-dimensional structure of the protein is perceived and defined by means of a description of the geometric structure of the polypeptide chain expressed as secondary and super-secondary structures. Proteins are characterized by various forms: helical, β-structural, sandwich, propeller, and others as super-secondary forms and various quaternary forms. Discussing the structural diversity of proteins is one of the basic topics of biochemistry textbooks [1]. In the initial period of operation of the Protein Data Bank (PDB) [2], each subsequent structure appearing in the collections of this database was treated as a source of information on the structure of proteins. After several years of PDB operation, it turned out that the structures consist of similar systems built of helices and β-beta sheets differing only in their mutual orientation. However, there were forms referred to as ‘new folds’—structures revealing surprising conformations mainly at the level of super-secondary structure [3,4]. In the CASP (Critical Assessment Structure Prediction) project, the new fold category was introduced to determine the uniqueness of a given structural form, where homology-based methods are not applicable [5,6]. Visual analysis of a large number of proteins, however, still evaluates their diversity as surprisingly high.

The basic element taken into account in numerous methods oriented on predicting 3D structure is the process of optimization of internal interactions, which results from the spontaneous nature of the folding process and from the possibility of reversibility of the folding process of proteins [7].

The assumption that the structure is encoded in the amino acid sequence raises however some doubt especially since the identification of the so-called chameleon sequences [8]. Fragments with identical sequences of 6–9 amino acids represent various forms, including extreme: helical in one protein and β-structural in another. It can be concluded that the structure depends on the surrounding of the given fragment and on the entire remaining part of the given protein [9]. The number of proteins in the database collecting the chameleon sequences is constantly increasing [10].

An additional surprising phenomenon is the observation of diametrically different structures in the presence of very few mutations [11]. A classic example is de novo generated proteins, where in a chain of 56 amino acids, 3 mutations result in a conversion of a helical form into a beta-structure [11]. An additional extreme example is proteins where a single mutation in a chain of 56 amino acids generates diverse structural forms described as 3α and 4β + α [12,13]. The introduction of the term “metamorphic” to describe this state of affairs seems to be justified [11]. The proteins mentioned so far are de novo designed. The construction of such proteins is precisely aimed at revealing significant differences in the spatial structure of the protein under the influence of very small sequence differences. However, it turns out that, in living organisms, proteins with “metamorphic” structures are also observed [14,15,16].

The problem of metamorphic proteins can be extended to protein structures that show structural variation regardless of the presence of mutations [17,18,19,20]. Very often, structural changes are associated with protein complexing to specific target systems including the cell membrane in particular [21].

External factors are also taken into account as affecting structural changes in proteins [22,23,24,25]. Oligomerization is also recognized as a phenomenon that stimulates the adoption of different structural forms of metamorphic proteins [26].

The reference to chameleon proteins with structurally diverse sections (fragments) of the polypeptide chain with an identical amino acid sequence length—e.g., nine amino acids—suggests the entire chain is involved in the structure of the native structure. The element stabilizing the tertiary structure is (in addition to disulfide bonds) the presence of a hydrophobic core. The hydrophobic core understood as the concentration of high-hydrophobic amino acids in the center and the exposure of hydrophilic residues on the surface requires the cooperation and involvement of almost all amino acids in a joint action that occurs at the level of the entire molecule and not a single amino acid. We can treat such an action as a kind of synergy, where the components lead to the creation of a new quality which is the presence of a hydrophobic core. Its structure, in turn, can be compared to the structure of a spherical micelle, which arises spontaneously as a response to the presence of the water environment. The specificity of the water environment results in the construction of a system with an ideal spherical hydrophobicity distribution. Its structure is described by means of 3D Gaussian distribution. Analysis of protein structures with a very diverse secondary and tertiary structure based on the characteristics of the hydrophobic core using the fuzzy oil drop model (FOD) has proved the universality of the use of this model to assess the degree of orderliness in accordance with the 3D Gaussian distribution [27].

The issue of generating a structural form that is different from the biologically active one takes on a significant meaning in the case of the so-called misfolding diseases, where proteins form fibrillar forms with highly pathological significance [28,29,30,31].

Therefore, a question was asked regarding proteins with a low degree of sequence diversity (1 mutation per 56 amino acids), showing structural diversity of monomeric 3α and 4β + α folds, about the cause of this phenomenon based on the differences in the structure of the hydrophobic core [12,32].

Analysis of these proteins based on the characteristics of the hydrophobic core form reveals the possibility of alternative organization of the central core [33].

In the present study, the object of analysis is proteins with a minimal degree of sequence diversity: 1 mutation in a chain of 57 amino acids, which results in structure diversity 3α and 4β + α [12]. The analysis is based on the fuzzy oil drop model [34].

The use of the fuzzy oil drop model allows for the assessment of the presence of an alternative hydrophobic core in the case of amyloids, leading to the formation of band micelle forms in place of the spherical micelle, which guarantees the solubility of individual protein molecules [35].

The aim of the present work is to show the possibility of the presence of different forms of the hydrophobic core as an effect of specific synergy, which, depending on the sequence (1 mutation in the chain of 56 amino acids) leads to different forms of protein. The analysis was carried out based on the use of the fuzzy oil drop model and the model for determining the structure of the early intermediate in the process of folding the polypeptide chain.

## 2. Materials and Methods

### 2.1. Data

The subject of the analysis are the proteins listed in Table 1 where their brief characteristics are also given. The proteins listed in Table 1 are de novo design proteins. The goal of the synthesis of these proteins is to show the effect of a single change in the sequence resulting in a change in the entire organization of the monomeric 3α and 4β + α folds molecule, which is also associated with the change in the secondary structure for individual chain sections.

### 2.2. Early Stage Model—Structural Codes

The early stage model was described in detail in [33,36,37,38,39]. To make the interpretation of results easier the basic elements of this model is given.

**Figure 1:**
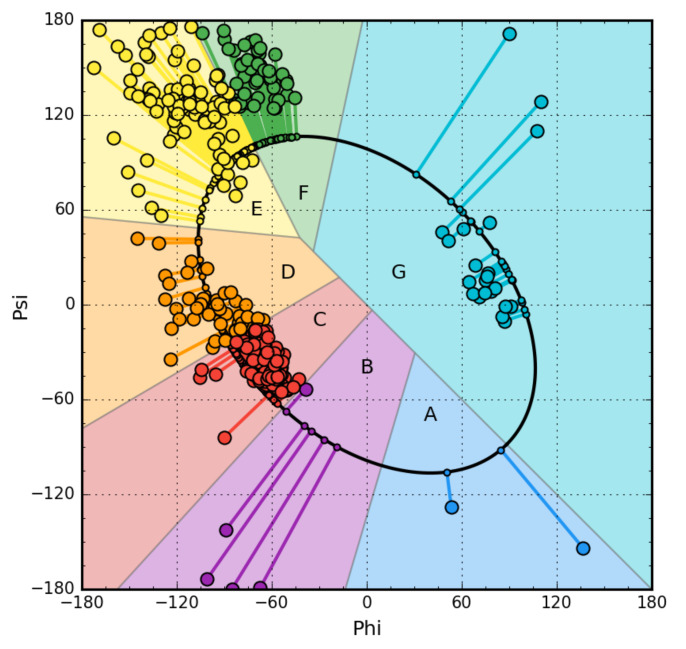
Example of Phi and Psi angle map for chain A from NAD-dependent formate dehydrogenase from higher-plant *Arabidopsis thaliana* in a complex with NAD and azide (PDB ID: 3N7U) [40]. This protein has been selected due to its large size (351 aa per chain), allowing sufficient representation of secondary structure elements in all structural code zones. The lines represent the transformation of Phi and Psi into Phi_e_ and Psi_e_ forms.

The starting point is the analysis of the backbone structure in five-alanine peptides (Phi and Psi angles equal for all alanines in the peptide). For the generated structures covering the entire Ramachandran map with a 5-degree step, two parameters were determined: The radius of curvature R, which the structure reveals, and the angle of opening between the planes of two adjacent peptide bond planes (by what angle should the peptide bond plane be rotated around the Cα-Cα axis to get the orientation of the analogous plane in the neighboring bond) called V-angle (the effect of Phi and Psi rotations). The radius for the α-helix is known in the literature (this is given by almost every biochemistry textbook) and is a small value. The V-angle of the helix is close to zero because all peptide bond planes point in a common direction (if the peptide bond plane is given as a vector according to the polarity system). The β-structure represents a form with a very large radius of curvature at a V-angle close to 180 degrees (peptide bond planes rotated in opposition to each other). The dependence of the radius R on the angle V shows the form of a parabola, which means that the intermediate forms are obtained by gradually increasing the V-angle (the effect of Phi and Psi rotation), which results in an increase in the radius of curvature. If we assume that the determined dependence indicates optimal geometrical conformations for the backbone, then it is possible to identify an area on the Ramachandran map that exactly meets the conditions of the discussed dependency. It turns out that this is an elliptical path connecting all low-energy secondary forms. Because this path meets the condition of representing all secondary forms, it was assumed that it represents the so-called limited conformational sub-space for the early intermediate in the process of folding the polypeptide chain (Figure 1). The transformation of Phi, Psi angles present in native structures into the Phi_e_ and Psi_e_ form (the index “e” means belonging to the elliptical path, i.e., limited conformational sub-space) reveals the distribution of Phi, Psi angles throughout the map. The distribution of points on the elliptical path reveals the presence of seven local maxima. Each of them identifies the map area with increased concentration of Phi, Psi angles. That is why individual zones are distinguished on the map, whose A–G codes are also identifiers of the type of pentapeptide conformation. This distribution was determined on the basis of a non-redundant protein set [36].

The introduced letters (Figure 1) denote the system of structural codes resulting from the distribution of angles in native proteins. The code C specifies the right-hand helical structure, the code G the left-hand helix. The codes E and F identify structures traditionally referred to as β-strands. However, it turns out that the analysis of the area referred to as β-structure shows a clear differentiation in the form of two local maxima. The E code specifies the extended forms observed in β-structured sections. In contrast, the code F represents the status corresponding to the terminal fragments of β-forms with a reduced radius of curvature, which always accompanies to terminate the extended form.

Using the discussed model, any structure can be determined using structural codes. A set of codes was determined for the proteins discussed in this paper. They were used to identify structural differences that are not disclosed in the secondary-structure classification. The extended presentation of early stage structure is discussed in the detailed presentation is given in Supplement A.

### 2.3. Fuzzy Oil Drop Model Expressing the Structure of a Hydrophobic Core

This model has been described many times in publications (it is fully discussed in [41,42,43]). Here it is presented to a degree that allows correct interpretation of the results presented here. The detailed presentation is given in Supplement B.

It is assumed that the structure of the protein (especially globular, and such proteins are the objects of the current analysis) can be represented by an ellipsoid (3D Gaussian distribution) stretched so that the entire molecule is encapsulated. The ellipsoid size is expressed by appropriately selected σX, σY, and σZ values. The value of the function at any point i-th within this ellipsoid (as assumed) expresses the idealized level of *Ti* hydrophobicity.

On the other hand, however, one can determine the contribution of individual residues in the structure of the hydrophobic core by determining the level of hydrophobicity resulting from the specific distribution of residues in the protein and the interaction between them, referred to here as Oi. The magnitude of this interaction depends on the intrinsic hydrophobicity of each residue as well as the distance between them. To determine the real status of a given residue, taking into account the immediate environment of a given residue, the function proposed in [44] was used. Each residue is represented by the so-called ‘effective atom’—the average position of all heavy atoms contained in a given amino acid. This point is assigned the value of *Ti* and *Oi.* The distribution of *Oi* and *Ti* values after their normalization (the sum of all *Oi* and *Ti* is equal to 1. respectively) is ready for comparison. The degree of matching the *O* distribution to the *T* distribution can be determined based on Kullback–Leibler divergence entropy *D_KL_* [45]. However, the value determined in this way cannot be interpreted directly because of the status of this quantity as an expression of entropy. Therefore, a second reference distribution is introduced (the first is the *T* distribution with an ideally centric hydrophobic core) in the form of a unified distribution referred to as *R*, where the level of hydrophobicity for each residue is 1/N where N is the number of amino acids in the protein. The reference distributions: *T* and *R* represent two opposing states. In the first, the core is perfectly centric and the level of hydrophobicity decreases according to the bell curve in each direction as it moves away from the center reaching values close to zero on the surface (i.e., at a distance of 3 times σ for each direction). In the second case, the molecule is devoid of any differentiation in the level of hydrophobicity, representing a constant level regardless of location. Determining by means of *D_KL_* the status of the entire molecule in relation to two distributions allows determining the degree of similarity of the *O* distribution to the *T* distribution and the R distribution. The status of the *O* distribution is determined on the basis of the smaller “distance” from the reference distribution. This type of assessment requires the use of two values. To avoid this, the so-called relative distance (*RD*) defined as shown below has been introduced

(1) $$RD=\frac{D_{KL}(O|T)}{D_{KL}(O|T)+D_{KL}(O|R)}$$

where *D_KL_*(*O|T)* is the value of *D_KL_* for the relationship of the distribution *O* to the distribution *T*, while *D_KL_(O|R*) is the value of *D_KL_* for the relationship of the distribution *O* to the *distribution R*.

An *RD* value less than 0.5 means the presence of a central hydrophobic core (O distribution being closer to *T* than *R* in terms of *D_KL_*).

With the help of the fuzzy oil drop model, it becomes possible to identify residues that are part of the hydrophobic core based on high *Oi* values and high *Ti* values for a given residue. On the same principle, it is also possible to identify residues located on the surface (low *Ti* and low *Oi*). Residues showing incompatibility at large *Ti* disproportions to *Oi* are identified as introducing local disorder and thus weakening the action of the hydrophobic nucleus as a factor stabilizing the secondary structure. It should be noted that the interpretation of the concept of a hydrophobic core encompasses the entire protein, of course including the high-hydrophobic center but also the outer mantle in the form of a polar layer. The compatibility and synergy of all these elements covering the entire molecule qualifies a given protein as more or less stabilized by the presence of a hydrophobic core.

The analysis based on the fuzzy oil drop model is presented in this work, where the causes of the radically different structure and composition of the hydrophobic core in proteins with a minimal degree of sequence differences are analyzed. The structure of the hydrophobic core in proteins differing in a single mutation leads to a change in the structure from the monomeric form 3α and 4β + α in the following proteins: Ga98, Gb98 (L45Y versus Ga98), and Gb98-T25I and Gb98-T25I,L20A where T25I and L20A mutations were introduced in addition to L45Y. These proteins are de novo design proteins. Their synthesis was aimed at checking the possibility of converting the 3α structure into the 4β + α form by using only a single mutation [11,12,13,32].

**Figure 2:**
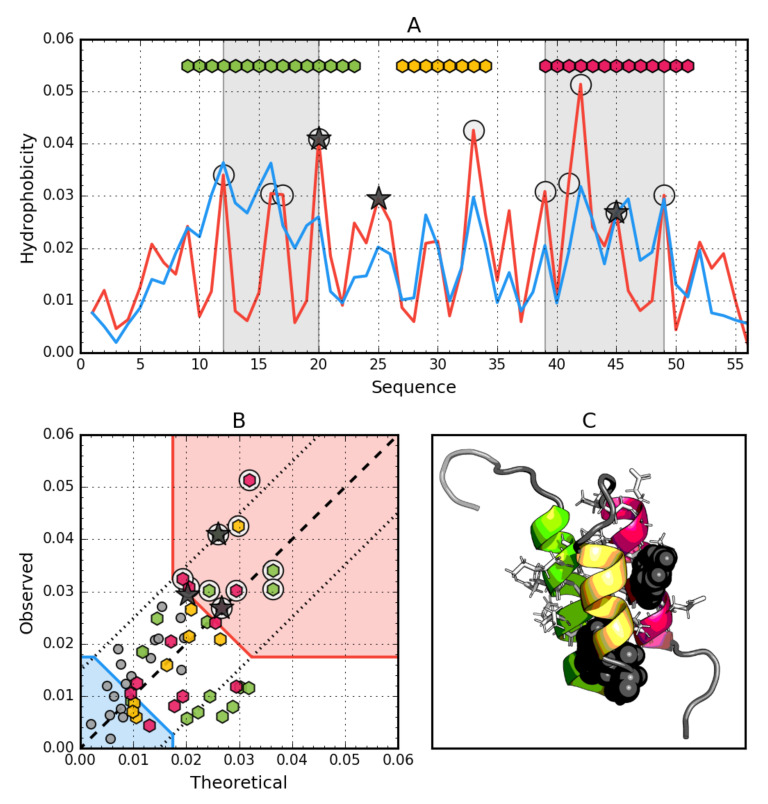
Hydrophobic properties of Ga98 (2LHC). (**A**) theoretical (*T*—Blue) and observed (*O*—Red) hydrophobicity density profiles. Markers at the top denote individually colored elements of the secondary structure: Hexagons—α-helices, squares—β-strands. White circles mark the location of hydrophobic core as determined by the FOD model. Gray-shaded regions encompass contiguous sequence fragments containing this core. Black stars locate the mutated positions in the other three proteins (20L, 25T, 45L). (**B**) *T* and *O* hydrophobicity density scatter plot. Red shade—hydrophobic core zone, blue shade—hydrophilic surface zone. Markers denote the same residues as markers on A, except for small dark gray circles which are hydrophobicity parameters of unstructured fragments. Dashed line—*T* and *O* value equality line. Dotted lines—*T* and *O* value similarity threshold. (**C**) 3D presentation of the molecule. Secondary structure are distinguished using the same colors as on A. Hydrophobic core fragments are shown as white sticks, while mutations are shown as black spheres.

In addition to comparing *RD* values or individual proteins and the status of their fragments with a specific secondary structure, the analysis of the relationship between *Ti* and *Oi* was determined by determining the regression function Y = X (in our case *T* = *O*) as expressing the idealized status of residues included in the protein (dashed line in Figure 2B and other). The tolerance zone was determined on the basis of the *T* distribution quartiles. Residues between two straight lines parallel to the line *T* = *O*: Passing through the points [*T* = Q1, *O* = Q3] and [*T* = Q3, *O* = Q1] are considered as consistent with the model (low difference between *T* and *O*, dotted lines in Figure 2B and other). In addition, two straight lines parallel to the Y = −X line passing through the points [*T* = Q3, *O* = Q3] and [*T* = Q1 and *O* = Q1] define respectively the hydrophobic core zone (high *T* and high *O*, red shade in Figure 2B and other) and hydrophilic surface zone (low *T* and low *O*, blue shade in Figure 2B and other). Residues in the central rhombus cut from the *TO* space by these four lines denote hydrophobically insignificant residues—those that both fill the space between core and surface and express no ligand binding or P-P interaction properties.

For a full description, the correlation coefficient between the *T* and *O* distribution was also calculated for whole particles and for selected fragments.

Programs used for graphic presentations are: PyMOL [46] and Matplotlib [47].

## 3. Results

For a protein with a hydrophobic core structure consistent with Gaussian 3D distribution, a linear relationship between *Ti* and *Oi* is expected. A function of type *T* = *O* means idealized compatibility. Any deviation from this relation indicates the degree of deviation from the idealized state of the given residue. When the difference between *T* and *O* becomes too large, compatibility replaces incompatibility, suggesting a possible ligand binding pocket [48] or protein–protein interaction area [49]. Therefore, the subject of analysis is the relationship between *Ti* and *Oi* in these proteins.

### 3.1. Ga98 (2LHC)

The data given in Table 2 indicate that the whole molecule represents the presence of a highly ordered hydrophobic core with a centric location (*RD* < 0.5). In addition, the status of individual helical sections is highly consistent with the idealized state, especially helix 2, where a very high correlation coefficient is also observed.

Particular mention should be made of position 45L, which according to the representation in Figure 2. is located in close proximity to the function *T* = *O*, i.e., the values of *Ti* and *Oi* are very similar. This means that 45L fits very well in the construction of the central hydrophobic core.

Interpretation of highlighted areas in Figure 2: residues representing low *Ti* and *Oi* values indicate the status of residues exposed on the surface (highlighted in Figure 2B with a blue shade). These residues correctly carry out the task of building a hydrophilic coating on the surface. The area marked as red shade in Figure 2B shows high *Oi* values as expected. Residues belonging to this area are components of the hydrophobic core. Their status is also as expected. The area between the areas in question represents an intermediate zone, where residues also represent the status as expected.

On the basis of Figure 2A,B, residues building up the hydrophobic core can be identified (marked with circle markers). Figure 2 shows the amphipathic nature of the helices, whose *Oi* distribution is matched to the expected Ti distribution.

The spatial arrangement of the residues included in the hydrophobic core and the outer shell shown in Figure 2C shows the very good organization of the entire molecule from the point of view of the structure of the hydrophobic core.

The graph in Figure 2A,B shows a strong hydropathic character of this helix, where the values displayed on the surface show a much lower level of hydrophobicity (high level of hydrophilicity) than would result from the location of these residues. This effect is quite lower for helix 1, however it has no influence on the entire molecule status.

### 3.2. Gb98 (2LHD)

**Figure 3:**
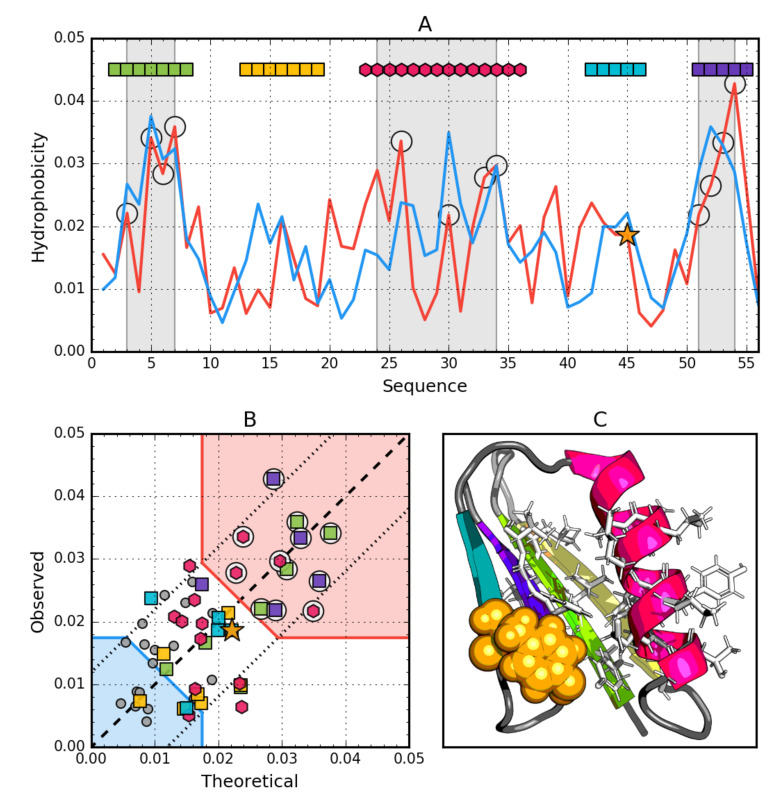
Hydrophobic properties of Gb98 (2LHD). (**A**) theoretical—blue and observed—red hydrophobicity density profiles. Markers at the top denote individually colored elements of the secondary structure: Hexagons—α-helices, squares—β-strands. White circles mark the location of hydrophobic core as determined by the FOD model. Gray-shaded regions encompass contiguous sequence fragments containing this core (combined with Gb98-T25I,L20A core data). Orange star locates the mutation (L45Y). (**B**) theoretical and observed hydrophobicity density scatter plot. Red shade—hydrophobic core zone, blue shade—hydrophilic surface zone. Markers denote the same residues as markers on A, except for small dark gray circles which are hydrophobicity parameters of unstructured fragments. Dashed line—Theoretical and observed value equality line. Dotted lines—theoretical and observed value similarity threshold. (**C**) 3D presentation of the molecule. Secondary structures are distinguished using the same colors as on A. Hydrophobic core fragments are shown as white sticks, while mutation as orange spheres.

Mutation L45Y changes in the form of an increase in the *RD* value for the entire molecule compared to the 45L version, although the status expressed with *RD* < 0.5 means the presence of a central hydrophobic core. As many as three β-structured fragments have an *RD* value higher than 0.5, which is significant especially for sections 12–20 and 42–46. The segment containing the changed element 45Y (relative to Ga98) shows even negative correlation, which means a significant deviation from the expected distribution. This does not mean, however, that the only reason is the status of residue 45 itself, because as one can see in Figure 3A,B, the status of 45Y exactly matches the expectations of idealized distribution.

The status of the complete β-sheet, however, turns out to be consistent with the distribution expected from both the point of view of the *RD* value (<0.5) and the relatively high correlation coefficient (Table 3).

**Figure 4:**
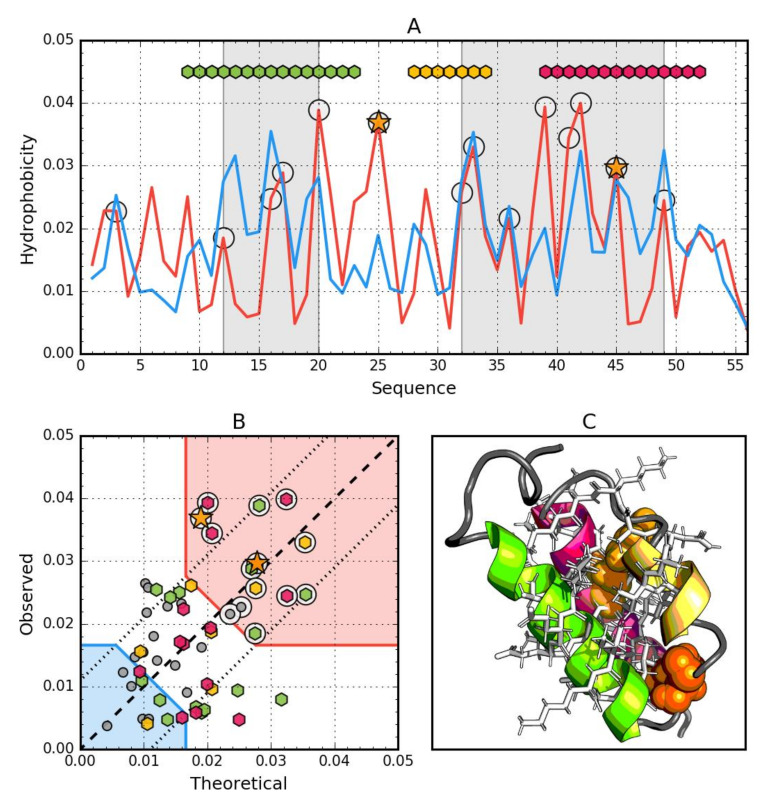
Hydrophobic properties of Gb98-T25I (2LHG). (**A**) theoretical—blue and observed— red hydrophobicity density profiles. Markers at the top denote individually colored elements of the secondary structure: Hexagons—α-helices, squares—β-strands. White circles mark the location of hydrophobic core as determined by the FOD model. Gray-shaded regions encompass contiguous sequence fragments containing this core. Orange stars locate the mutations (T25I, L45Y). (**B**) theoretical and observed hydrophobicity density scatter plot. Red shade—hydrophobic core zone, blue shade—hydrophilic surface zone. Markers denote the same residues as markers on A, except for small dark gray circles which are hydrophobicity parameters of unstructured fragments. Dashed line—theoretical and observed value equality line. Dotted lines—theoretical and observed value similarity threshold. (**C**) 3D presentation of the molecule. Secondary structures are distinguished using the same colors as on A. Hydrophobic core fragments are shown as white sticks, while mutations as orange spheres.

The analysis of *T* and *O* distributions (Figure 3A) and the relation from Figure 3B observed in Gb98 suggest a very high consistency of both compared distributions. In Figure 4B, virtually no residue remaining outside the compliance area is observed. The status of the Beta 2, Beta 3 and H segment identified by *RD* assesses these sections as representing some degree of non-compliance locally.

However, the profiles (Figure 3A) reveal a fundamental difference in the composition of the hydrophobic core. The N-terminal segment showing a very high level of *T* and *O* hydrophobicity and similarly the C-terminal seem to be important building blocks for the construction of the hydrophobic core. This phenomenon is not seen at all in the graphs in Figure 3A, where involvement in the structure of the core comes from helical segments rather in the middle of the chain.

The helical segment—present in both proteins in question—plays a similar role as a component of the hydrophobic core.

### 3.3. Gb98-T25I (2LHG)

The data given in Table 4 indicate that the whole molecule represents the presence of a highly ordered hydrophobic core with a centric location (*RD* < 0.5). In addition, the status of individual helical sections except helix 8–24 is consistent with the idealized state, especially helix 2, where a very high correlation coefficient is also observed similarly to Ga98.

Particular mention should be made of position 45L, which according to the representation in Figure 3, is located in close proximity to the function *T* = *O*, i.e., the values of *Ti* and *Oi* are very similar. This means that 45L fits very well in the construction of the central hydrophobic core.

Interpretation of highlighted areas in Figure 4: Residues representing low *Ti* and *Oi* values indicate the status of residues exposed on the surface (highlighted in Figure 4B with a blue shade). Residues contained in the highlighted area correctly carry out the task of building a hydrophilic coating on the surface.

The area marked as red shade in Figure 4B: the residues show high *Oi* values as expected. Residues belonging to this area are components of the hydrophobic core. Their status is also as expected. The area between the areas in question represents an intermediate zone, where residues also represent the status as expected.

On the basis of Figure 4A,B, residues building up the hydrophobic core can be identified (marked with circle markers). Figure 4 shows the amphipathic nature of the helices, whose *Oi* distribution is matched to the expected *Ti* distribution.

The spatial arrangement of the residues included in the hydrophobic core and the outer shell shown in Figure 4C shows the very good organization of the entire molecule from the point of view of the structure of the hydrophobic core.

The graph in Figure 4A,B shows a strong hydropathic character of this helix, where the values displayed on the surface show a much lower level of hydrophobicity (high level of hydrophilicity) than would result from the location of these residues. This effect is quite lower for helix 1, however it has no influence on the entire molecule status.

### 3.4. Gb98-T25I, L20A (2LHE)

The Gb98-T25I,L20A protein among those discussed so far has the highest degree of agreement of the *O* distribution against the *T* distribution, which is expressed by the lowest value of both *RD* and correlation coefficient for the complete molecule (Table 5). The status of the beta sheet present in this protein is also highly compatible, although one of its fragments (42–46) shows (similar to Gb98) a status with a relatively high *RD* value (exceeding the 0.5 threshold) and a negative correlation coefficient. Beta-structured sections (1–9 and 50–55) appear to represent the state as expected. The list of introduced mutations is given in Table 5.

It should be noted that the protein in question in relation to the second representative of the same class in the secondary structure (4β + α folds) shows sequence differences at two positions (20 and 25). Therefore, this is an example of obtaining almost identical structure despite the presence of two mutations. The change from Leu to Ala (position 20) does not bring a radical change in the level of hydrophobicity. However, the change of Ile to Thr (position 25) from the point of view of the distribution of hydrophobicity is a significant change. However, if the local environment does not change, this example indicates a local agreement that does not affect the status of the entire protein molecule from the point of view of the structure of the hydrophobic core.

**Figure 5:**
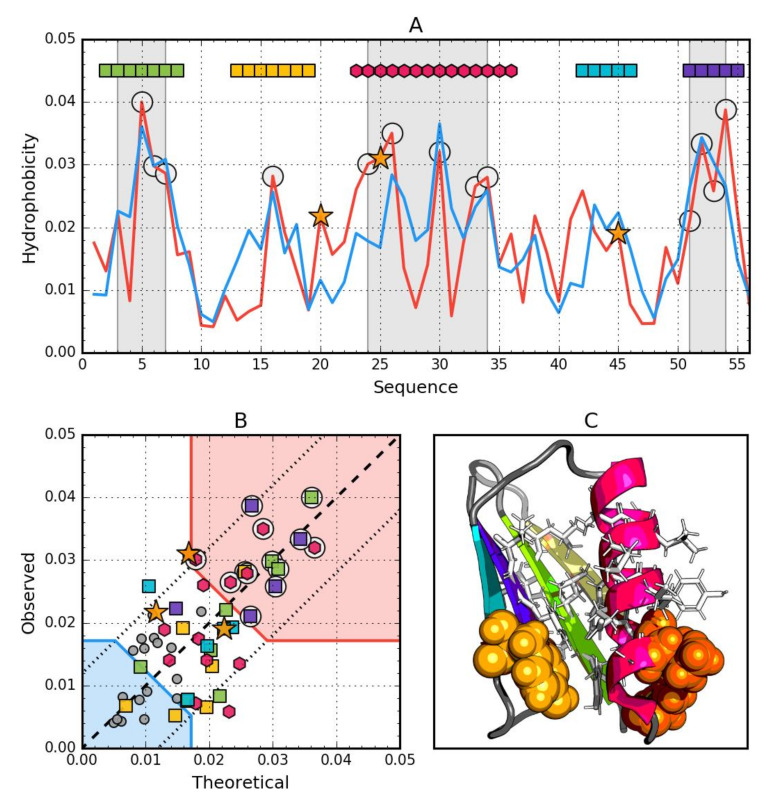
Hydrophobic properties of Gb98-T25I,L20A (2LHE). (**A**) theoretical—blue and observed—red hydrophobicity density profiles. Markers at the top denote individually colored elements of the secondary structure: Hexagons—alpha helices, squares—beta strands. White circles mark the location of hydrophobic core as determined by the FOD model. Gray-shaded regions encompass contiguous sequence fragments containing this core (combined with Gb98-L45Y data). Orange star locates the mutations (L20A, T25I, L45Y). (**B**) theoretical and observed hydrophobicity density scatter plot. Red shade—hydrophobic core zone, blue shade—hydrophilic surface zone. Markers denote the same residues as markers on A, except for small dark gray circles which are hydrophobicity parameters of unstructured fragments. Dashed line—theoretical and observed value equality line. Dotted lines—theoretical and observed value similarity threshold. (**C**) 3D presentation of the molecule. Secondary structure are distinguished using the same colors as on A. Hydrophobic core fragments are shown as white sticks, while mutations are shown as orange spheres.

The interpretation of the profiles (Figure 5A) reveals the involvement in the construction of the hydrophobic core clearly similar to the Gb98-L45Y protein. However, comparing the correlation values once RD, it turns out that the Gb98-T25I, L20A protein represents a status very close to a spherical micelle.

### 3.5. Comparative Analysis

The summary of *T* profiles for the four proteins Ga98, Gb98, Gb98-T25I, and Gb98-T25I, L20A reveals different expectations regarding the location of the hydrophobic core.

**Figure 6:**
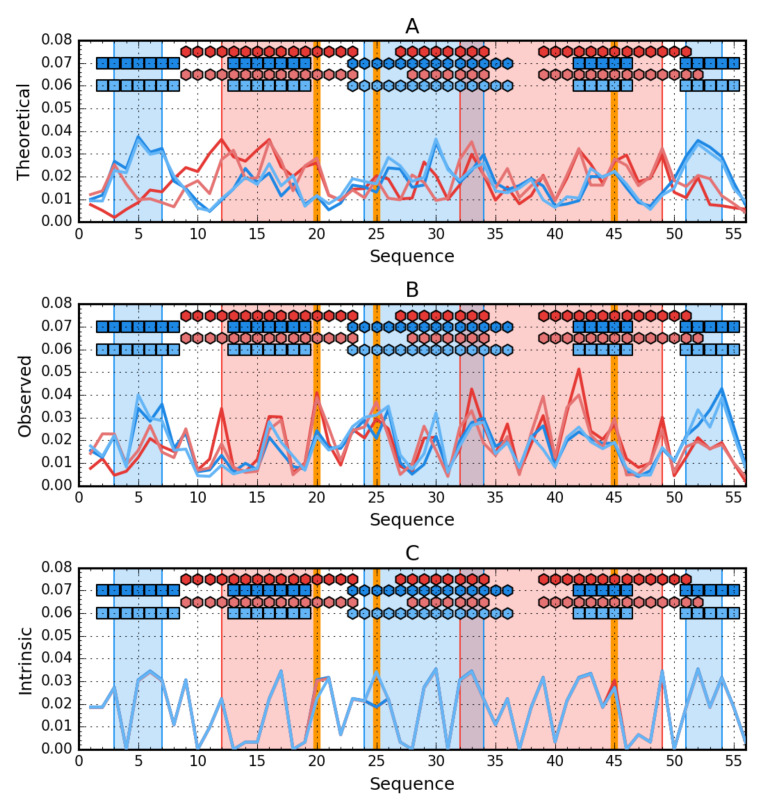
Hydrophobicity density comparison between Ga98 (red), Gb98 (blue), Gb98-T25I (bright red) and Gb98-T25I, L20A (bright blue). (**A**) theoretical (*T*) distributions; (**B**) observed; (*O*) distributions; (**C**) intrinsic (H) distributions. Markers at the top of each subplot denote elements of the secondary structure: Hexagons—alpha helices, squares—β-strands (Ga98 & Gb98-T25Y—Red, Gb98 & Gb98-T25I, L20A—Blue). Blue-shaded regions encompass contiguous sequence fragments containing hydrophobic core in Gb98 and Gb98-T25I, L20A. The cores in Ga98 and Gb98-T25I are shaded red. Mutations (L20A, T25I, L45Y) are shown as vertical orange lines.

Profile analysis in Figure 6 reveals the relationship between the hydrophobicity distribution for a β-structural form. Yellow frames very intuitively indicate positions that should be involved in the core structure showing high hydrophobicity. If we add the form of periodicity of changes in the level of intrinsic hydrophobicity and the overlap of the composition of the central helix with the periodicity expected for the amphipathic helix, the participation of segment 25–35 in the structure of the core in the form of a helix appears to be predictable. However, the presence of sections 9–13 and 46–50 in the structure of the hydrophobic core is surprising due to the low level of intrinsic hydrophobicity at this point.

The composition of the hydrophobic core for the 4β + α form surprises at position 9, which has not been incorporated into the core despite high intrinsic hydrophobicity.

The change in amino acid at position 25 apparently had no effect on the structuring of the helix. On the other hand, the exchange of L for A at position 20 could have affected the elimination of this position in the participation in the construction of the hydrophobic core. The change at position 45 in the form of a decrease in hydrophobicity created conditions for the exposure of section 46–50 in the form of 4β + α in the form of an external loop.

**Figure 7:**
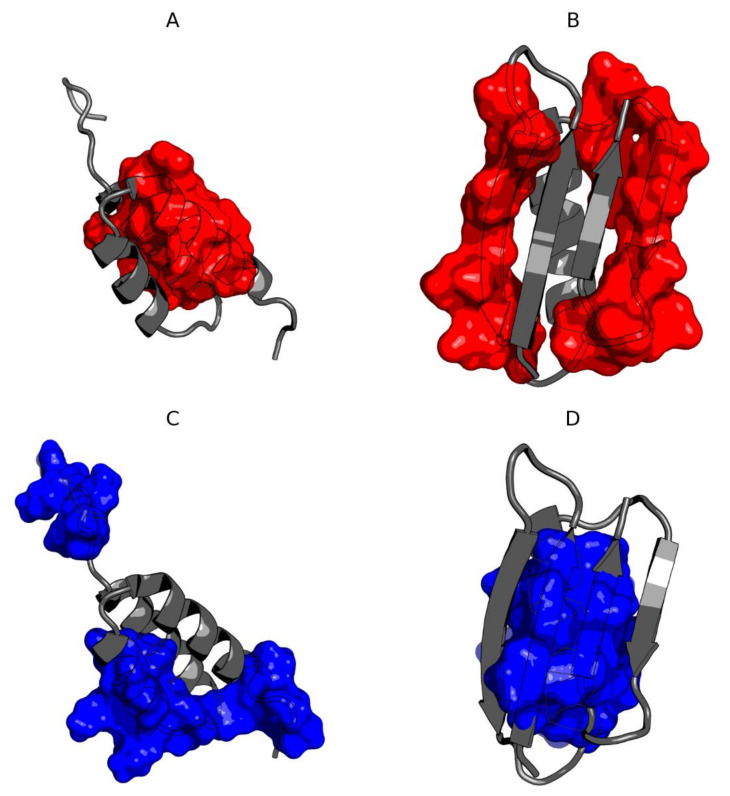
3D presentation of Ga98 (**A**,**C**) and Gb98 (**B**,**D**) showing significant differences in the location of hydrophobic core. (**A**) Members of hydrophobic core of Gb98 (12–20, 39–49) in Ga98; (**B**) Members of hydrophobic core of Ga98 (12–20, 39–49) in Gb98; (**C**) Members of hydrophobic core of Ga98 (3–7, 24–34, 51–54) in Ga98; (**D**) Members of hydrophobic core of Gb98 (3–7, 24–34, 51–54) in Gb98.

The composition of the hydrophobic core residues is different in both compared molecules except for those present in the central helix (Figure 7). The residues shared by both hydrophobic cores for both molecules are 32L and 42V. The immediate vicinity of 33I in Ga98 and 34A and 35A in Gb98 can also be included in the common hydrophobic core building region. The remaining residues that are essential components of the hydrophobic core come from completely different chain positions. Residue 45L coordinate the hydrophobic surrounding in Ga98. Individual residues, including 45L, were coordinated around the hydrophobic environment in Ga98. However, in Gb98, where this 45L jumper is absent, hydrophobic residues entered a different coordination system.

**Figure 8:**
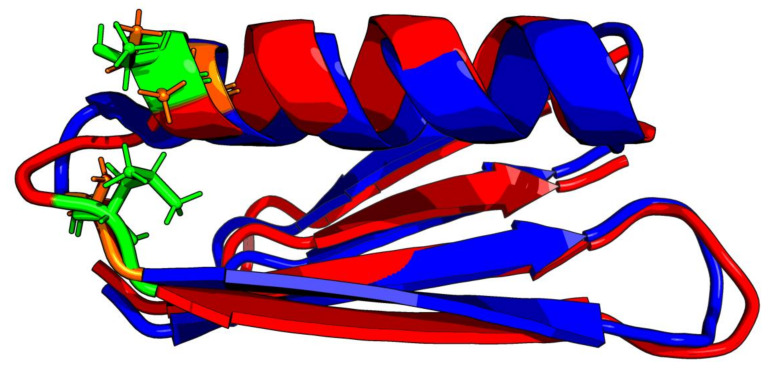
3D presentation of structural superposition of Gb98 (red) and Gb98-T25I, L20A (blue). RMSD = 1.9Å. Mutated residues are additionally shown as sticks: Green—20L and 25T in Gb98, orange—20A and 25I in Gb98-T25I, L20A.

Comparative analysis of Gb98 and Gb98-T25I, L20A does not provide significant information on the role of the amino acid at position 25. The location of these residues within the loop only shows loop tightening in the case of L20 (Figure 8).

### 3.6. Structural Codes to Express the Structural Changes

**Figure 9:**
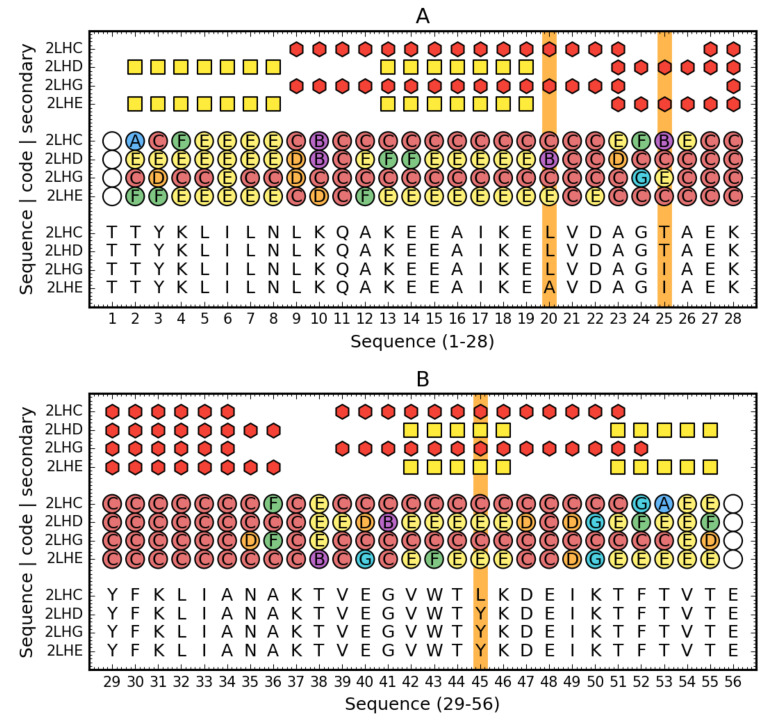
Comparison of Ga98 (2LHC), Gb98 (2LHD), Gb98-T25I (2LHG) and Gb98-T25I,L20A. Top rows: Secondary structure (red hexagons—helix, yellow square—sheet) for three compared proteins. Central rows: Structural codes (names and colors match the system used on Figure 1). Bottom rows: Sequence. Vertical orange line denotes the mutated residues. For readability, sequence is split into two parts (1–28—**A**, 29–59—**B**).

The description of the structure of these proteins based on the structural codes discussed in Materials and Methods reveals differences probably resulting from the presence of mutations (Figure 9).

The overall assessment of the composition of the hydrophobic core indicates a much greater degree of dispersion of the core-forming residues in the case of the 3α form, while the involvement in the structure of the 4β + α core concerns three clearly distinguished fragments: N-terminal, central, and C-terminal. The differentiating factor is position 45, where the presence of Leu, which is part of the core and at the same time is part of the helical segment, seems to play a major role. Position 25, despite varying levels of hydrophobicity in both cases, is part of the hydrophobic core. This position is important due to the fact that the presence of Thr did not affect the decision to choose a secondary structure.

The presence of the β-structure in the 3α form in positions 5–8, which did not enter the beta-sheet system with the C-terminal section, is puzzling. The low content of E forms in the C-terminal segment could have determined the lack of this type of interaction with the N-terminal segment. This may be due to the presence of 45Tyr, which is apparently surrounded by E-conformation residues in the 4β + α form.

The low involvement of the central helical segment in the structure of the 3α nucleus is also significant, whereas the situation in 4β + α indicates the important role of this helix in the structure of the hydrophobic core.

The conclusion from the analysis of this part of the results also relates to the fact that the direction of folding towards obtaining the 3α or 4β + α form takes place at a position in the C-terminal fragment (position 45). It can be concluded that the structure of the chain during the synthesis takes very low packing forms and the collapse associated with the formation of the hydrophobic core takes place only at the final stages of the folding process, which is also demonstrated by the great involvement in the construction of the hydrophobic core of the terminal chain residues (51–55).

## 4. Discussion

A common feature of the proteins discussed here is the high compatibility of observations with the model of loops connecting individual secondary fragments. In most cases, they are loops located in the surface zone, showing a low level of hydrophobicity of both *O* and *T*. This is obviously related to the assessment of the symmetry and ordering of the hydrophobic core. The whole protein molecule shows the compliance status of the *O* and *T* distribution. This means that the proportion of hydrophilic to hydrophobic residues guarantees the construction of a surface that is large enough for the size of the hydrophobic core. Disruption of this proportion in the form of an insufficient number of polar residues results in the appearance of local maladjustments to the spherical micelle.

Structural changes determine biological activity, as evidenced by metamorphic proteins, whose number is constantly increasing [50].

It has also been shown that a “metamorphic” change requires a significant degree of protein unfolding undergoing structural transformation [51]. The diversity of intermediates in the folding process in the context of evolutionary changes supports the conclusions regarding metamorphic transitions [52].

The dogma of structure determination by the amino acid sequence is perfectly confirmed in the case of the proteins in question. However, if you limit the analysis of the structure only to its geometric form—secondary structure—Then the exchange of one amino acid in the sequence of 56 amino acids resulting in the change of the helical form to β-structural is surprising. This surprise lasts as long as the fact that the 3D structure goes through the hydrophobic core stage is not taken into account. This quest to generate a hydrophobic core is the primary goal, and secondary structural forms are only a means to obtain a state with a hydrophobic distribution close to that observed from the spherical micelle. Proteins that do not show the presence of a hydrophobic core (centric concentration of high hydrophobicity) obtain it due to the presence of numerous disulfide bonds or complexation with the object (membrane or other organelle) in a permanent manner. A polypeptide chain with a specific sequence is able to produce a spherical micelle structure to varying degrees. The amino acid sequences in the proteins discussed here show this possibility, although the path and structural means leading to it are different.

Polypeptide chains with an amino acid sequence that excludes the possibility of forming a central core in terms of hydrophobicity distribution in the form of 3D Gaussian distribution show smaller or larger deviations from the idealized state. Local maladjustment—as it turns out—is very often associated with biological function. The essence of protein folding as a structure carrying information that determines biological function lies in the appropriately coded inability to generate a hydrophobic core in the sense of spherical micelle. Hence the term protein as an intelligent micelle that, not representing the perfect order, “knows” what function it will achieve through this lack of order [41].

In conclusion, it can be stated that the protein structure is the result of a specific synergy, where the pursuit of building a micelle type structure (in terms of 3D Gauss hydrophobicity distribution) can be implemented in various ways. In each case, the cooperation takes a form suitable for the distribution of hydrophobic residues in the chain.

It is puzzling that position 45 shows a perfect match to the *T* distribution (the *Oi* and *Ti* statuses of this amino acid are virtually identical) regardless of which amino acid occupies this position (Figure 2, Figure 3, Figure 4 and Figure 5). The more visible here is the role of synergy in the folding process and not the role of the individual residue as decisive for folding. The role of a single residue is to influence the overall synergy phenomenon and not to local ordering. Local disorder is treated as a carrier of information defining the biological activity of a given protein [53]. In this context, protein folding boils down to ensuring such local disorder that results in the quest for an optimal state. It can occur, for example, by binding a specific ligand, which in a specific way makes a given ‘local disorder’ closer to an optimal state. The group of proteins meeting the condition of ordering in accordance with the distribution of spherical micelles is type II antifreeze proteins, where perfect matching to the micelle structure ensures the solubility desired in this context [54].

The analysis presented here is closely related to the search for the mechanism of the amyloidogenesis process, where there is no mutational change and the structural transformation applies to the entire system, which is a single chain seeking to form oligomeric structures.

The difference in synergy in the generation of a hydrophobic core is also treated as a factor not only differentiating protein structures but also leading to the formation of an amyloid structure [54].

## 5. Conclusions

The work shows that the 3D structure of a protein as encoded in the amino acid sequence of a given polypeptide is the result of some specific synergy, which, taking into account the characteristics and physicochemical properties, produces a new quality in the form of a higher degree organization, which is the cooperative hydrophobic core. It, in turn, is the result of the interaction of individual amino acids and the aquatic environment, which directs the folding process towards a more or less perfect spherical micelle. The role of single mutations, especially changing the level of hydrophobicity, results in the participation of different residues in the formation of the hydrophobic core. The participation of three sections: central, N-, and C-terminal in the case of Gb98-T25I, L20A, and two fragments in Ga98 located between the above-mentioned fragments results in a different structure: 4β + α folds in the first and 3α in the second. The hydrophobic core is an effect of synergy in which the entire chain participates. The status of individual residues is the result of their own predisposition to participate in the formation of the core but also depends on the influence of the environment. The change in distribution favoring helix amphipathicity and beta-specific amphipathicity are decisive for adopting secondary forms. In the fuzzy oil drop model, the primary mechanism is to create a hydrophobic core, while the secondary structure is a means leading to this goal. To make the discussion of the presented problem compete the Ref. [55,56,57,58,59] are recommended.

## Figures and Tables

**Table 1:** Proteins being the object of analysis in the present work. Their short characteristics are also given.

Name	PDB ID	Mutation Position vs. Ga98	Chain Length	Structural Form	Source Organism	Ref
Ga98	2LHC		56 aa	3α	de novo	[11]
Gb98	2LHD	L45Y	56 aa	4β + α	de novo	[11]
Gb98-T25I	2LHG	L45Y, T25I	56 aa	3α	de novo	[11]
Gb98-T25I,L20A	2LHE	L45Y, T25I, L20A	56 aa	4β + α	de novo	[11]

**Table 2:** List of parameters characterizing the status of the whole Ga98 protein and the highlighted sections representing a specific secondary structure. Bold—*RD* status different from idealized (discordant fragment).

Ga98	Fragment	RD	Corr. Coeff.
Complete mol. Helix 1 Helix 2 Helix 3 Rest Helices together	1–56 9–23 27–34 39–51	0.428 **0.511** 0.166 0.365 0.287 0.426	0.550 0.344 0.919 0.648 0.811 0.496

**Table 3:** List of parameters characterizing the status of the entire Gb98 protein and the highlighted sections representing a specific secondary structure. Bold—*RD* status different from idealized (discordant fragment).

Gb98	Fragment	RD	Corr Coeff
Complete mol. Beta 1 Beta 2 Helix 1 Beta 3 (L45Y) Beta 4 Β-sheet Loops	1–56 1–8 13–20 23–36 42–46 50–55	0.452 0.301 **0.580** **0.531** **0.647** 0.445 0.411 0.486	0.588 0.746 0.028 0.228 −0.051 0.476 0.650 0.484

**Table 4:** List of parameters characterizing the status of the whole Gb98-T25I protein and the highlighted sections representing a specific secondary structure. Bold—*RD* status different from idealized (discordant fragment).

Gb98-T25I	Fragment	*RD*	Corr Coeff
Complete mol. Helix 1 Helix 2 Helix 3 (L45Y) Rest (T25I) Helices together	1–56 9–23 28–34 39–52	0.485 **0.509** 0.349 0.446 0.453 0.463	0.425 0.294 0.753 0.473 0.481 0.438

**Table 5:** List of parameters characterizing the status of the whole Ga98-T25I, L20A protein and highlighted sections representing a specific secondary structure. Bold—*RD* status different from idealized (discordant fragment).

Ga98-T25I,L20A	Fragment	*RD*	Corr. Coeff.
Complete mol. Beta 1 Beta 2 (L20A) Helix 1 (T25I) Beta 3 (L45Y) Beta 4 Β-sheet Loops	1–56 1–8 13–20 23–36 42–46 50–55	0.383 0.360 0.470 0.488 **0.686** 0.356 0.407 0.365	0.674 0.871 0.429 0.396 −0.252 0.683 0.670 0.684

## Supplementary Materials

The following are available online at https://www.mdpi.com/2218-273X/10/5/767/s1.
