# Supplementary material for: Structure of the Hydrophobic Core Determines the 3D Protein Structure—Verification by Single Mutation Proteins
Source: Biomolecules. 2020 May 14;10(5):767. doi: 10.3390/biom10050767 (PMC7281683; doi:10.3390/biom10050767)
Supplement: Supplementary file 1 [file biomolecules-10-00767-s001.pdf]

SUPPLEMENT A

Early Stage MODEL

The so-called early intermediate model was defined based on geometric analysis of the polypeptide chain structure taking into account only the preferences of a given amino acid to show a specific conformation resulting from the rotation of Phi and Psi.

Phi and Psi angles are a perfect and unambiguous form of encoding the structure of a given amino acid in a chain. The representation of the structure using the Phi and Psi angles, however, excludes – according to the Heisenberg uncertainty principle – the definition of the structure of the entire chain. However, if an outline of the curves of the chain is available (e.g. a ribbon-like presentation), it is not possible to find out the values of Phi and Psi angles that led to the creation of this particular form.

Therefore, a simplified geometric model was proposed, where the structure is expressed using two parameters: the radius of curvature (for the helix the concept of curvature and its value is available in every biochemistry textbook) and the so-called V-angle.

The parameter, which is the radius of curvature, can be generalized by revealing that the β- structure (or the so-called extended) representing the linear form of the chain can be described by a sufficiently large radius. Theoretically, the radius of curvature for a straight line is infinitely large.

Determination of the radius of curvature can be carried out for any structure by introducing a uniform orientation of the chain (say, a pentapeptide). If we orient the chain in space so that the average orientation of C = O bonds in this pentapeptide is consistent with the Z-axis, then the projection of the position of Cα atoms on the XY plane will allow the determination of the radius of curvature.

The second parameter – V-angle – requires the following explanation.

In the helix, all C = O bonds are unidirectional. If we assign a vector according to the orientation of its dipole to a C=O bond, it turns out that all such vectors in the helix are directed in the same direction. The V-angle value for the helix is zero degrees. This is because restoring the position of the next peptide bond does not need any rotation in the sense of the peptide bond plane deflection. This is due to comparable values of Phi and Psi angles for subsequent residues in the peptide.

For the β-strand structure, for which the Z-axis was also oriented in accordance with the orientation of the average C=O bond in the pentapeptide, it turns out that the transition from the position of the i-th amino acid peptide binding plane to i+1-th requires rotation by 180 degrees because in β-strand the orientation C=O is alternating.

Therefore, we already have two points: for the angle V = 0 the helix radius is low, for the angle V = 180 the radius is infinitely large (to eliminate manipulation with large values the ln(R) scale was introduced for R). The conclusion of this analysis is: the value of the angle V-angle is a simple consequence of the rotation of Phi and Psi, while the size of the radius of curvature is a simple consequence of the size of the angle V (the angle by which the plane of the adjacent peptide bond should be rotated around the Cα-Cα axis to obtain the orientation present in a given peptide). All intermediate forms in this situation must be from 0 to 180 degrees for the angle V and the size of the radius on a scale from ln(R) equal to about 0.5 for the helix to ln(R) = 11 for β-strand and extended structures.

This relationship can be determined using, for example, a tri-peptide. However, this is not a representative unit for all secondary forms. Therefore, a pentapeptide was used to also include β-turns, for example. The odd number of amino acids in the structural unit under analysis is due to the need for non-zero orientation of the C=O groups. The necessity to use a pentapeptide results also from the fact that only for a certain segment of the chain the determination of the radius of curvature makes sense.

Conducting an analysis of all possible conformations (complete Ramachandran map) with a step of angle change Phi and Psi = 5 degrees, determining the value of the discussed parameters indicates the relation between ln(R) and V-angle as taking the form of a parabolic function (Figure 1.B). The limitation of structures to energetically allowed areas (areas of the Ramachandran map with allowable energy states – Figure 1.A) reveals the exact form of this relationship.

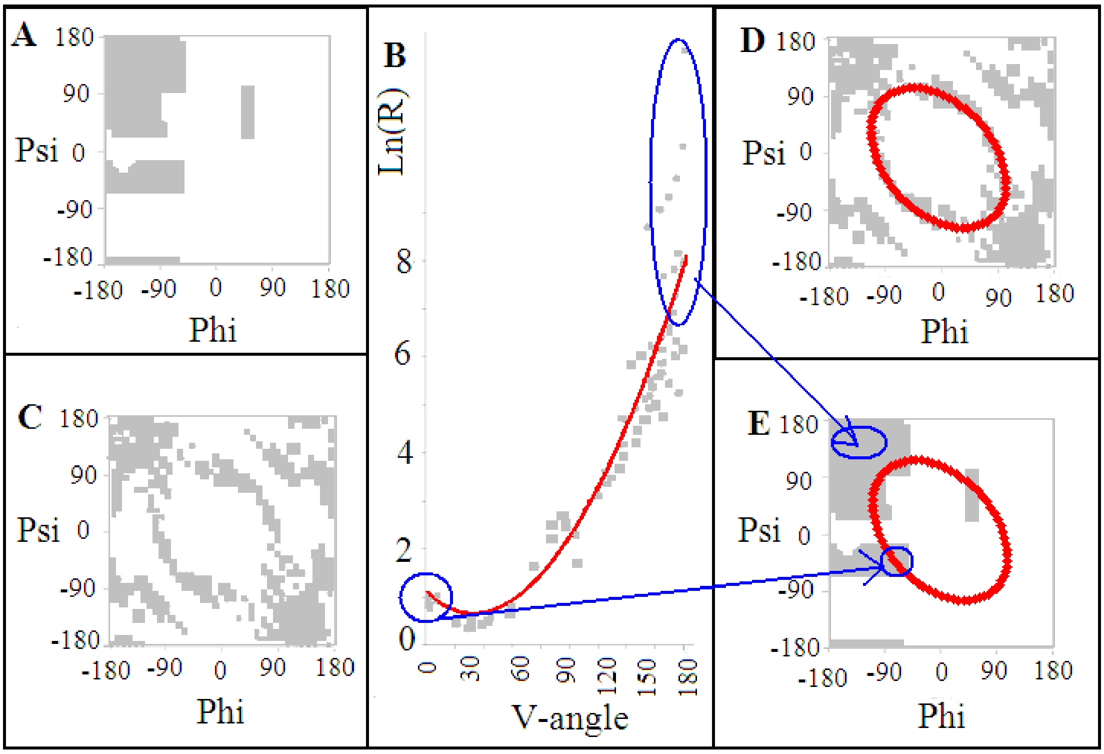

Figure AS1. Ramachandran map and the relation with V-angle and radius of curvature (R). **A** – low energy area. **B** – ln(R) dependence on V-angle with approximation function – red line. Values shown here are from the low-energy areas of the Ramachandran map with a 5 degree step for Phi and Psi angles. **C** – distribution of structures meeting the relation set out in B. **D** – elliptical line determined by the approximation method. **E** – d elliptical path in relation to low-energy areas revealing the passage of the elliptical path through areas representing secondary structures. This is shown by the blue arrows from B to E.

The next step is to look for those points (those conformations) that meet the designated relationship in a manner consistent with it (Figure 1.C). The distribution of these points on the Ramachandran map takes the form of an ellipse (Figure 1.D) with a very valuable feature: it connects all areas representing the so-called secondary forms.

It should be noted that this path results from the analysis of the optimal relationship between the rotations Phi and Psi (V-angle) and the resulting radius of curvature (Figure 1.B). Moving along the ellipse, we travel smoothly from the helix to the β-strand and then to the left-handed helix. Moving along the ellipse guarantees the availability of all secondary forms (Figure 1.E).

This path results from the preferential relationship of the V-angle and the curvature that the given V-angle value generates. Each Ramachandran map showing the distribution of Phi and Psi angles for a sufficiently large protein suggests just such a path of structural changes.

Adopting the conducted reasoning enables the analysis of conformational preferences of individual amino acids.

Phi, Psi angles present in proteins (non-redundant base of protein structures deposited in PDB [36]) after transformation into their Phi_e and Psi_e counterparts (index e means belonging to the elliptical path – Phi_e and Psi_e values determined on the basis of the criterion of the least subordination between Phi and Psi and the belonging points to the ellipse) reveal different distributions for subsequent amino acids. Generally, the presence of seven local maxima is determined by the corresponding letter codes A-G.

Codes: C are helix, E and F for traditionally defined β-structured form. In our model, the presence of two local maxima (Figure 2.A) differentiates the form E (a typical β-strand) and F, which is attributed to the conformation ending a straight segment of β-strand by introducing a stop turn protecting unlimited linear propagation. The F code indicates a slight bend indicating the end of the β-strand form. Analysis of profiles for individual amino acids reveals their preferential differences. All profiles are shown in [36]).

The most interesting code is the code D, which is an intermediate region between helix and β- structure. Handling a pentapeptide with fixed Phi and Psi angles does not limit the model. It is only an idealized structure. Calculation of the radius of curvature as well as the values of V-angle can be performed for any

section of the peptide from any protein. Such analysis allows the identification of positions that more or less apply the discussed model.

Phi_e and Psi_e angles determined according to the early intermediate model change significantly as a result of non-binding interactions with the remaining chain fragments, which the model does not take into account.

In the model discussed here only the orientations of the peptide bond planes are taken into account. Non-binding interactions change conformation towards the location of energy minima resulting in the appearance of final (active) values of the Phi and Psi angles [36-39].

The protein structure – as it is assumed – in the early form of the folding process – can be determined by providing a set of letter codes defining belonging to a given ellipse fragment.

The complete non-redundant PDB protein base has been subjected to a change in the Phi and Psi angles to the Phi_e and Psi_e values [36]. The resulting distribution of probabilities of a given conformation reveals the presence of seven local maxima (Figure 2.).

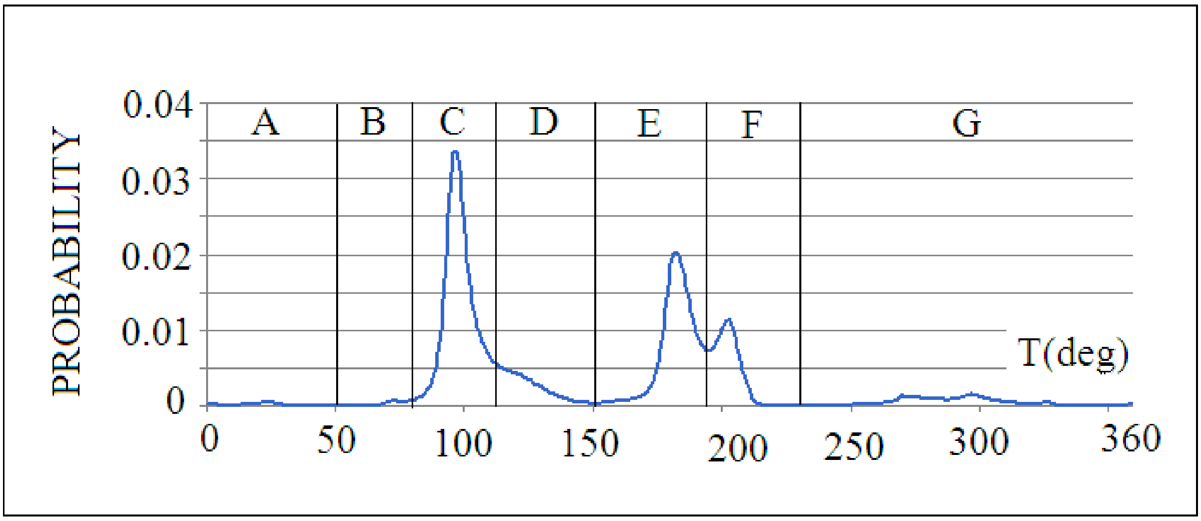

Figure AS.2. Probability profile of Phi_e and Psi_e pair matching given structural code along the ellipse. T(deg) is the T-angle for ellipse equation starting from the point 4.30 o'clock.

The reservations regarding the determination of R and V-angle parameters for a peptide with identical Phi and Psi angles can be easily explained. The pentapeptide was adopted so that the radius of curvature could be analyzed. For shorter peptides, this concept is not justified. The stability of the Phi and Psi angles is a form of system idealization. The V-angle calculation can be performed for any conformation within the pentapeptide by focusing on two central planes of peptide bonds. Such analysis allows to assess the presence of sections with the R versus V-angle system as relaxed in the case of identifying this relation as consistent with the parabola determined for the idealized system.

The set of structural codes for describing the differences present in the proteins discussed in the publication not only illustrates the hypothetical differences in early stage structures but is a symbolic record of the Ramachandran map area from which the angles Phi and Psi originate in the native form of the protein in question.

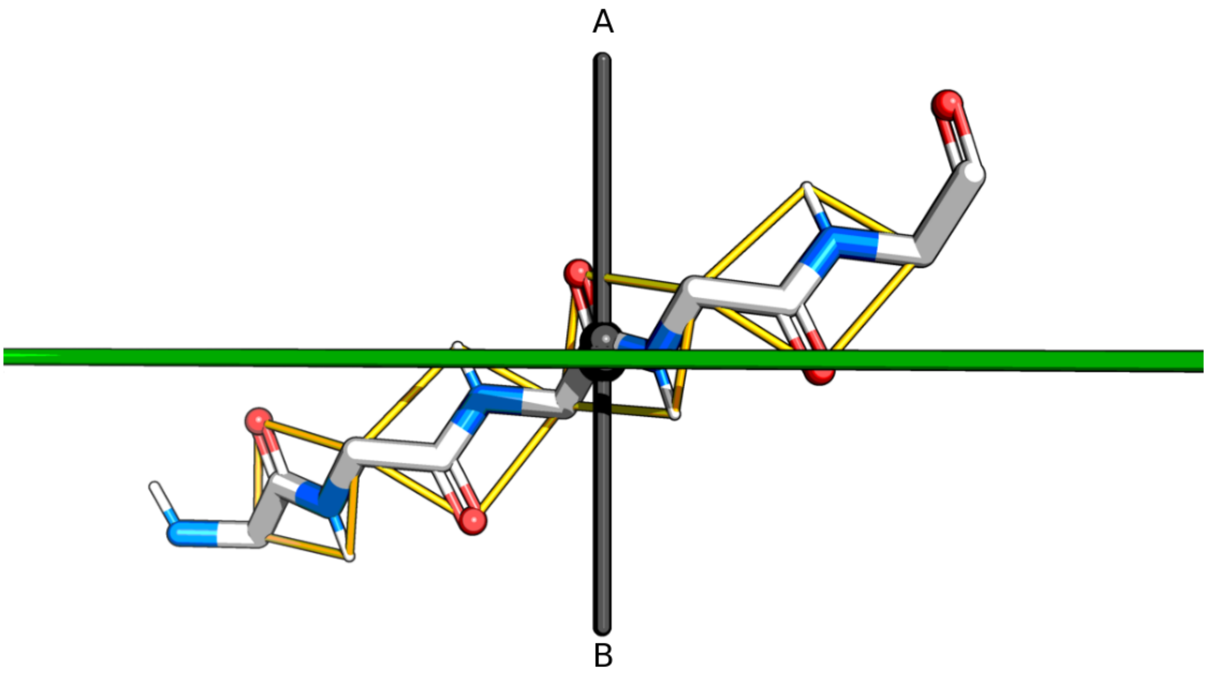

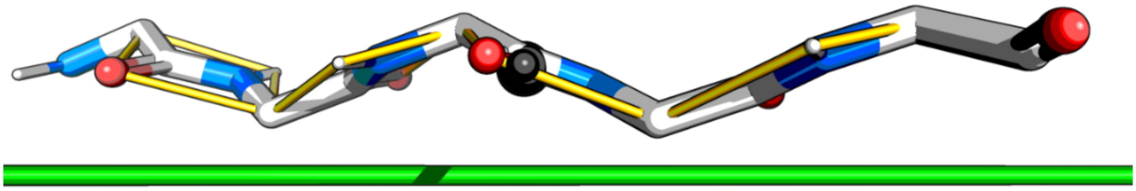

Figure AS.3. 3D presentation of early stage model calculation for the backbone of alanine pentapeptide in β-form. All dihedral angles have following values: Phi = -140°, Psi = 135°, omega = 180°. **A** – view on Z-axis from XY plane. **B** – view on XY plane from Z-axis. Yellow lines denote the peptide bond planes. Black line is the Z-axis with two black spheres marking the locations of average C-α atom (bottom sphere) and average oxygen atom (top sphere). Green line is an arc of a circle fit to positions of all C-α atoms projected onto the XY plane: ln(R) = 10.96.

Figure 3.A visualizes the mutual orientation of peptide bond planes in extended form o polypeptide the with the C=O bonds highlighted to show the 180 deg rotation expressed by V-angle. The peptide bond plane of i-the residues to reach the orientation of the i+1-th residue shall be rotated by 180 deg. Assuming the C=O orientation as the marker.

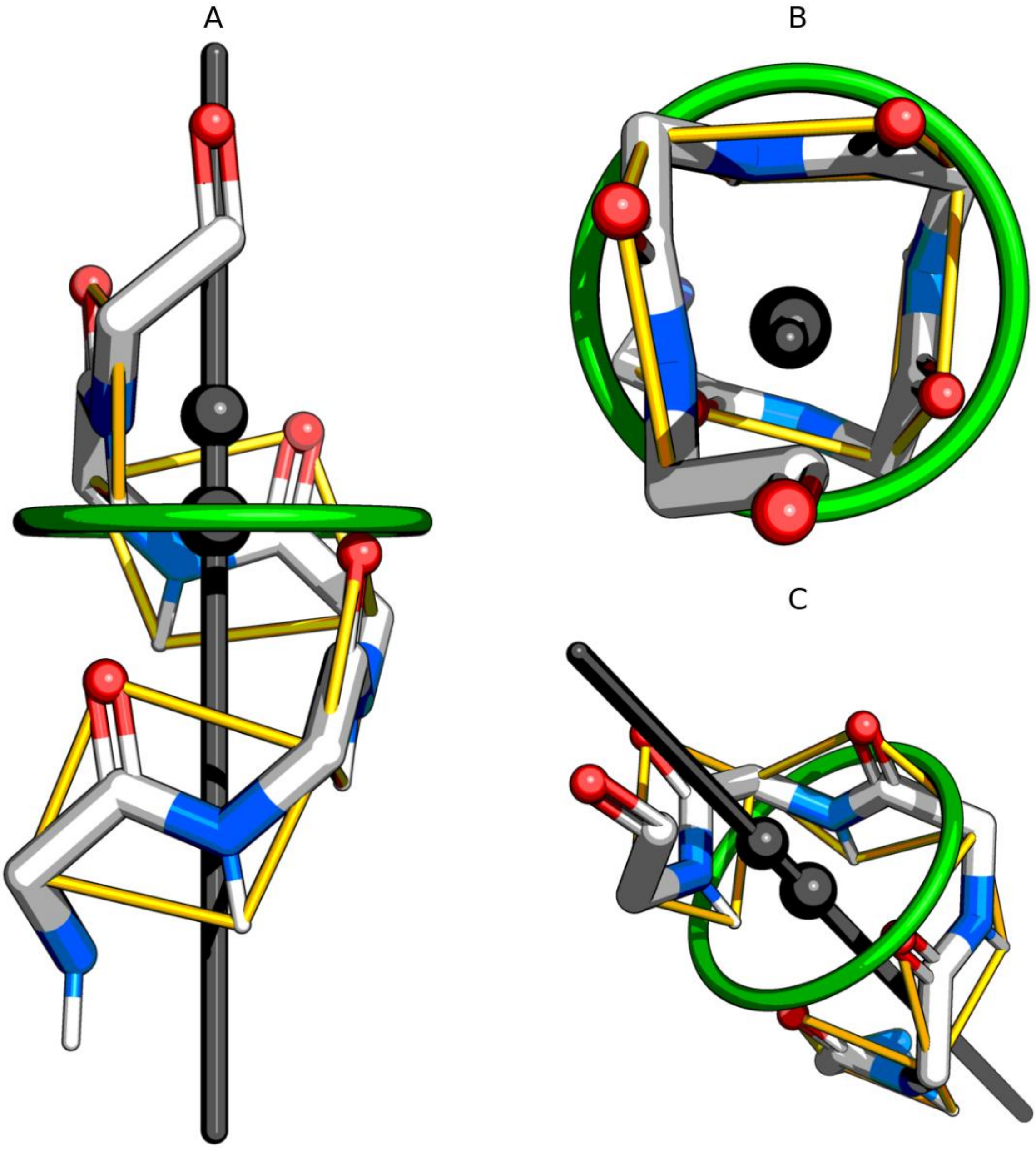

Figure AS.4. 3D presentation of early stage model calculation for the backbone of alanine pentapeptide in helical form. All dihedral angles have following values: Phi = -60°, Psi = -45°, Omega = 180°. **A** – view on Z-axis from XY plane. **B** – view on XY plane from Z-axis. **C** – perspective view. Yellow lines denote the peptide bond planes. Black line is the Z-axis with two black spheres marking the locations of average C-α atom (bottom sphere) and average oxygen atom (top sphere). Green circle is fit to positions of all C-α atoms projected onto the XY plane: ln(R) = 0.82.

Figure 4 visualizes the mutual orientation of peptide bond planes in helix the with the C=O bonds highlighted to show the V-angle close to 0 deg. No rotation is expected for one peptide bond plane to reach the orientation of the neighbors one assuming the C=O direction as the marker.

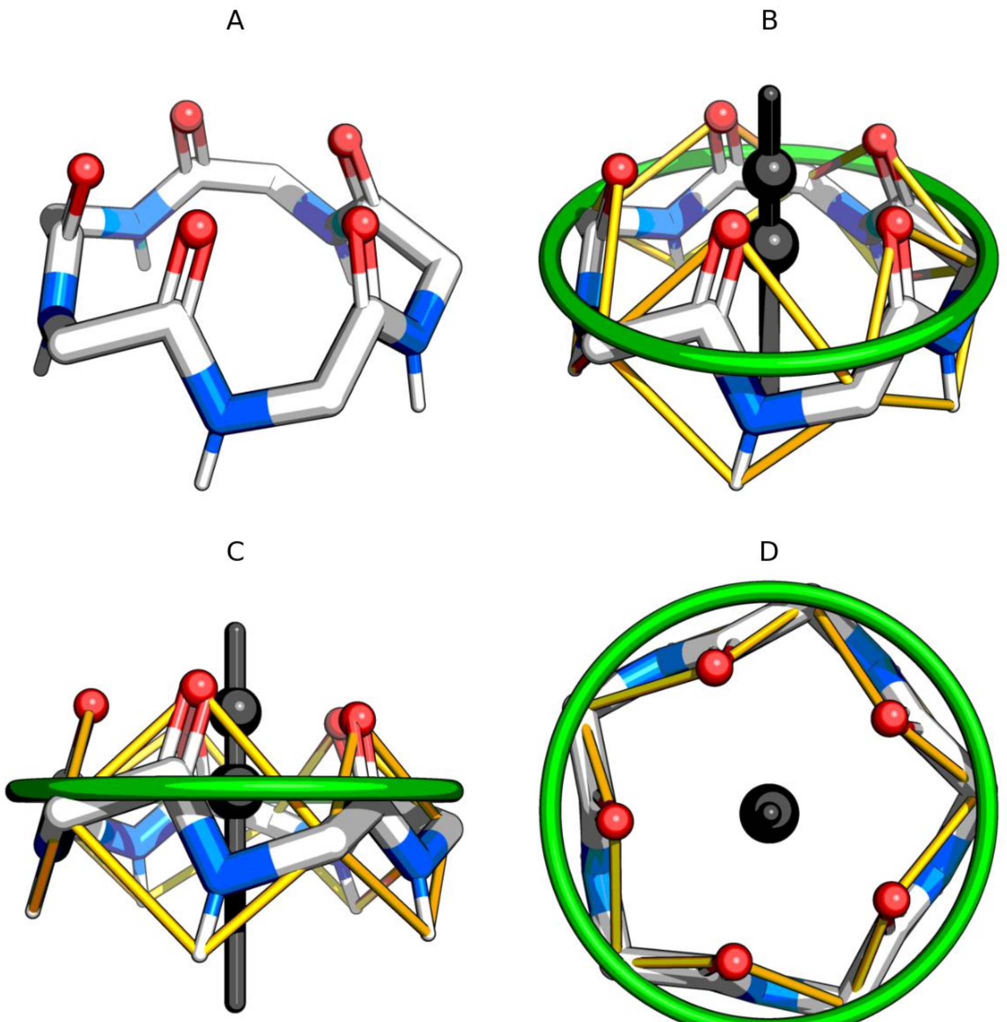

Figure AS.5. 3D presentation of early stage model calculation for the backbone of alanine cyclic pentapeptide. All dihedral angles have following values: Phi = -60°, Psi = -94°, Omega = 180°. **A** – general molecule view. **B** – perspective view. **C** – view on Z-axis from XY plane. **D** – view on XY plane from Z-axis. Yellow lines denote the peptide bond planes. Black line is the Z-axis with two black spheres marking the locations of average C-α atom (bottom sphere) and average oxygen atom (top sphere). Green circle is fit to positions of all C-α atoms projected onto the XY plane: ln(R) = 1.15.

Figure 5. visualizes the V-angle and R-radius of curvature as it is observed in cyclic-pentapeptide. Is is difficult to show the mutual orientation between two sequential peptide bond planes. This is why the secondary forms are selected.

SUPPLEMENT B

Fuzzy oil drop MODEL

The main assumption adopted in the definition of the fuzzy oil drop model is the conversion of the discrete form proposed by Kauzmann [55] into a continuous model. The original two-level model consisting of an outer layer of a polar character and a centrally located hydrophobic nucleus with high hydrophobicity called "oil drop" was replaced by a model called "fuzzy oil drop" model, where the level of hydrophobicity gradually decreases from the maximum in the center of the molecule to the minimum on its surface according to the bell curve of the Gauss function. A comparison of these two models in graphic form is illustrated in Figure 1.

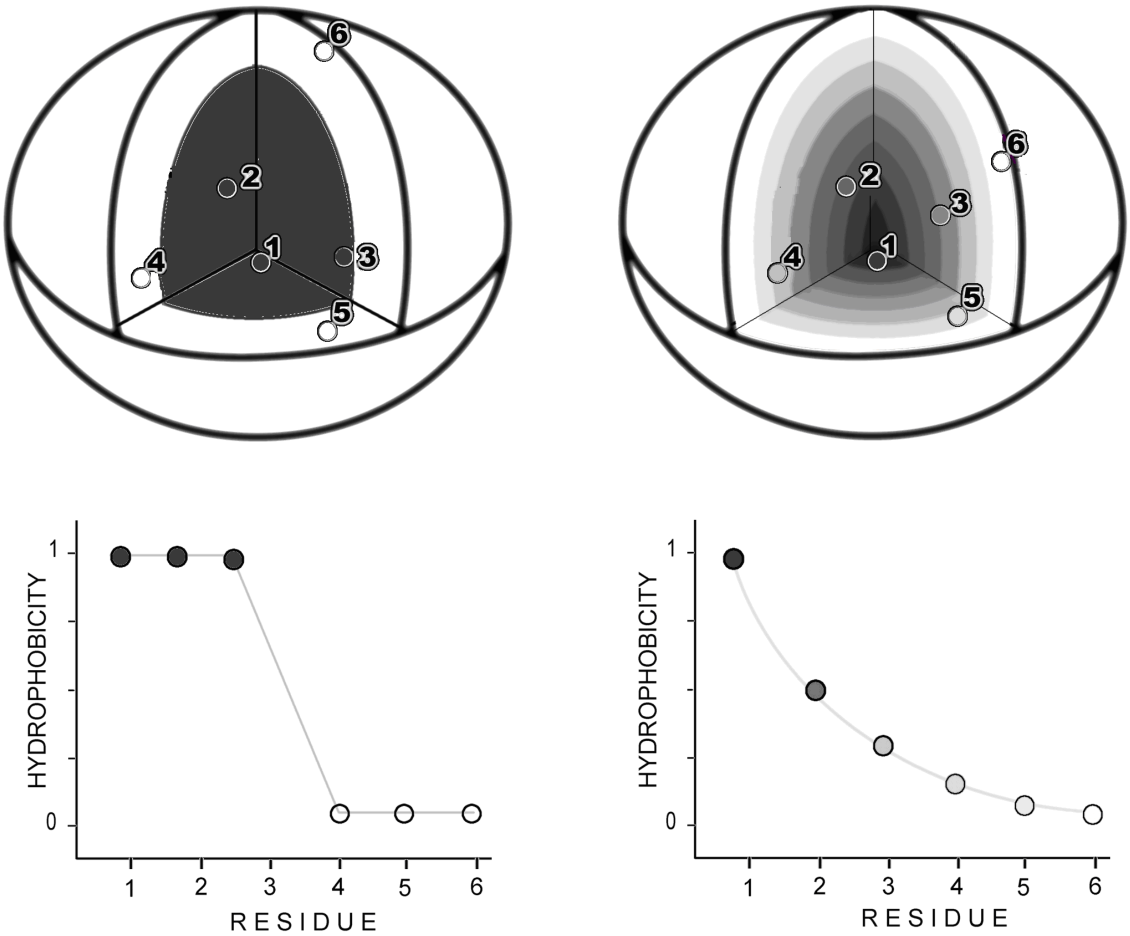

Figure BS.1. Distribution of hydrophobicity in a hypothetical protein molecule. Instead of a discrete black and white status: core / surface (left), a continuously-changing status is introduced by the fuzzy oil drop model (right). Residues harmonize their position with their intrinsic hydrophobicity and with the expected level of hydrophobicity in the protein body. Bottom profiles express the status of each residue in both models.

To represent the continuous nature of the hydrophobicity level changes according to the fuzzy oil drop model, a 3D Gaussian distribution was adopted to describe the hydrophobicity distribution representing the status present in the idealized globular micelle. It arises spontaneously from bi-polar molecules to form a spherical micelle. If two different bipolar molecules are present in the solution (with different proportions of the polar part to the hydrophobic part), it is possible to generate the so-called co-micelles made of these different molecules. However, the mechanism is common – the isolation of hydrophobic parts by means of a polar layer exposed on the surface.

The amino acid chain is nothing more than a set of 20 different bi-polar molecules with varying proportions of the polar part to the hydrophobic part.

The presence of a covalent bond imposing the order of amino acids introduces a significant reduction in the number of degrees of freedom and excludes the free selection of neighbors. Therefore, the polypeptide chain is unable to adapt the hydrophobicity distribution to the idealized micellar distribution.

A measure of the order of the hydrophobic core according to the rules of the spherical micelle can be determined using the procedure given below.

The protein molecule of any size is placed in the coordinate system so that the geometric center is at the point (0,0,0), the longest axis coincides with the selected axis (e.g. X). Then the molecule is oriented so that the longest segment connecting the points of projection of the positions of effective atoms (average positions of all heavy atoms in every residue) on the YZ plane coincides with the direction of the next axis (Y). Knowing the maximum distances to the point (0.0.0) along each axis, it is possible to determine the values of the 3D Gauss function parameters, which are the values σX, σY, σZ as 1/3 of the maximum distance (three sigma principle).

The notation of the 3D Gauss function used in the model is:

$$H_i^T = \frac{1}{H_{sum}^T} \exp\left(\frac{-(x_i - x)^2}{2\sigma_x^2} \right) \exp\left(\frac{-(y_i - y)^2}{2\sigma_y^2} \right) \exp\left(\frac{-(z_i - z)^2}{2\sigma_z^2} \right) \tag{1}$$

As a result, the protein molecule is encapsulated in an ellipsoid, which is a globular form of micelles allowing greater or smaller elongation along a specific axis.

The distribution determined with the help of 3D Gaussian function is defined as T (theoretical), assigning the status to a given residue as Ti - the value of 3D Gaussian function at the point of location of the effective atom of the i-th residue. The value of Ti is assumed to express the idealized hydrophobicity level in position of i-th residue (effective atom). H_{sum}^T is the normalization coefficient (sum of H_i^T). H_i^T shortly called later as Ti). However, due to the inability to generate the perfect micelle - as described above - another hydrophobicity distribution, the so-called Observed is analyzed: an actual distribution of hydrophobicity resulting from the location of individual residues in the protein body and their neighborhood. Here, Levitt's functions were used to describe the hydrophobic interaction [44], where the level of hydrophobicity of a given amino acid depends on its own intrinsic hydrophobicity and the distance between interacting residues. This distance is determined as the distance between the positions of their effective atoms. The simplification of this notation is justified by the characteristics of hydrophobicity as a characteristic of the whole amino acid.

The form of the Levitt function used [44]:

$$H_i^O = \frac{1}{H_{sum}^O} \sum_j \begin{cases} \left(H_i^r + H_j^r\right)\left(1 - \frac{1}{2}\left(7\left(\frac{r_{ij}}{c}\right)^2 - 9\left(\frac{r_{ij}}{c}\right)^4 + 5\left(\frac{r_{ij}}{c}\right)^6 - \left(\frac{r_{ij}}{c}\right)^8\right)\right), \text{for } r_{ij} \le c \\ 0, \text{for } r_{ij} > c \end{cases} \tag{2}$$

Where H_i^O denotes the experimentally observed (index O) hydrophobic density at particular point (position of effective atom of *i-th* residue) which collects the hydrophobic interaction in distance dependent form as given in the formula with the cutoff distance (c) assumed according to original work 9Å [44]. The denominator H_{sum}^O (sum of all H_i^O) makes the value in normalized form. H_i^r and H_j^r express the intrinsic hydrophobicity of *i-th* and *j-th* residues, which can be taken according to arbitrarily selected scale [34].

Using this function, the value of the so-called Oi is assigned: the observed level of hydrophobicity, which is the sum of interactions with neighbors. This means that the intrinsic hydrophobicity of a given amino acid undergoes modification due to the neighborhood and impact of that neighborhood on its hydrophobicity.

Both distributions: T and O after normalization (the sum of all Ti = 1 and the sum of all Oi = 1) can be compared quantitatively (normalization coefficients H_{sum}^O are included in formulas (1) and (2)).

Quantitative measurement of differences between T and O distributions is determined using a divergence entropy function introduced by Kullback-Leibler.

$$D_{KL}(P \| Q) = \sum_i P(i) \log_2 \frac{P(i)}{Q(i)} \qquad (3)$$

Where *P(i)* denotes the observed probability (hydrophobicity density) localized on i-th residue – O_i in this paper – and *Q(i)* which denotes the expected (target distribution) hydrophobicity localized on the same residue – T_i in this paper. D_{KL} therefore corresponds to the "distance" between O and T, the latter of which is regarded as the reference.

In a simplified form, the dependence of O on T may be written as:

$$O \| T = \sum_{i=1}^{N} O_i \log_2 (O_i / T_i) \qquad (4)$$

The value determined in this way cannot, however, be analyzed as an absolute measure of entropy. Therefore, a second reference distribution is introduced, opposite to the T distribution, in the sense that it represents a uniform distribution of hydrophobicity throughout the molecule. Each residue is assigned the same level of hydrophobicity equal to $Ri = 1 / N$ where N is the number of amino acids in the chain.

T distribution represents the presence of an idealized hydrophobic core, which is covered by a polar layer (hydrophobicity level close to 0), while R distribution excludes differentiation of hydrophobicity at any point in the protein. Therefore, these reference distributions represent exactly opposite systems.

The calculation of the D_{KL} "distance" between O and R is given by the formula:

$$O \| R = \sum_{i=1}^{N} O_i \log_2 (O_i / R_i) \qquad (5)$$

This allows to determine the difference between distribution O and T and R distributions. $O||T < O||R$ means that O is "closer" to T than to R while $O||T \geq O||R$ means that O is "closer" to R than to T.

To avoid using two values describing the status of a given protein, the RD (Relative Distance) parameter was introduced, expressed as follows:

$$RD = \frac{D_{KL}(O \| T)}{D_{KL}(O \| T) + D_{KL}(O \| R)} \qquad (6)$$

RD expresses therefore the relation between O and two other distributions – T and R. Unlike D_{KL}, this value (between 0 and 1) is independent of the length of the chain and may be used to characterize any protein. An RD value less than 0.5 indicates the presence of a hydrophobic core (same as $O||T < O||R$).

Graphically, this characteristic (reduced to one-dimensional presentation) is depicted in Figure 2. In this example, the interpretation of the results indicates a high similarity of the T and O distributions.

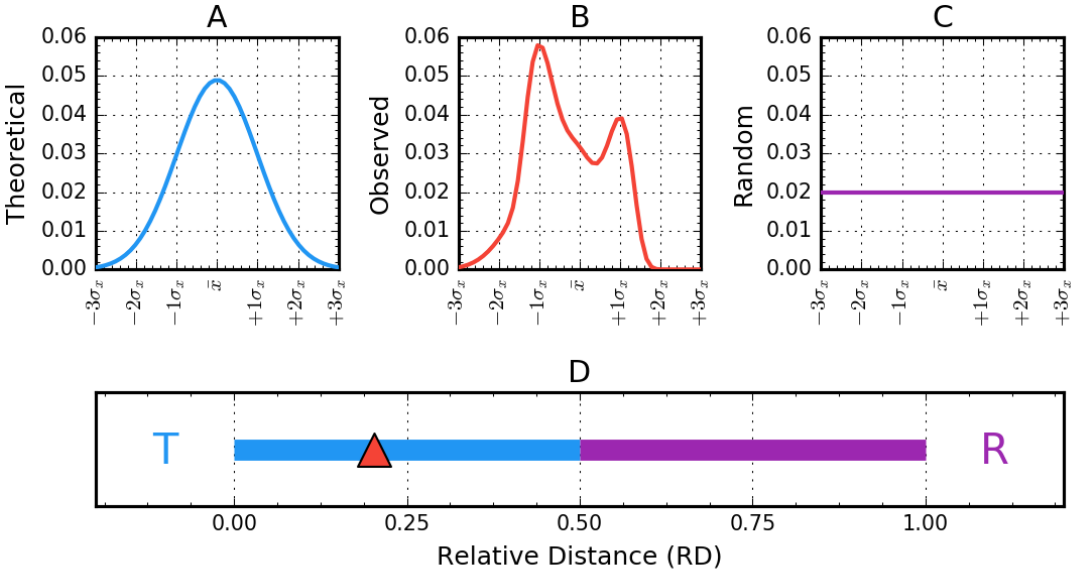

Figure BS.2. Examples of hydrophobicity distributions in a hypothetical, one-dimensional protein: **A** – idealized (T), **B** – observed (O), **C** – uniform (R), **D** – RD scale. Position of the red triangle visualizes the value RD = 0.202, which denotes that a hydrophobic core is present in this molecule (O||T < O||R).

Analysis of the protein structure based on the fuzzy oil drop model identifies proteins from the point of view of the presence of a hydrophobic core, which, apart from disulfide bonds, is treated as a factor stabilizing the tertiary structure. Therefore, the RD value can be interpreted as a kind of measure of the stabilization of the molecule.

The structure of the hydrophobic core in accordance with micellar decomposition results in the properties of the protein as very soluble and not interacting with other than water. Is such a protein needed at all by the cell? It turns out that such proteins are responsible for performing a critical biological function. Proteins meeting the high compatibility of the T and O distribution have been identified. This is a whole group of proteins – antifreeze type III proteins. They are small proteins present in hibernating organisms. The process preventing the formation of ice structure consists here in imposing the ordering of water molecules in a way resulting from the distribution of polar groups on the surface of these proteins and thus excluding the possibility of ordering the ice present in the structure. The Na^+ and Cl^- ions spilling out into the streets in winter do the same. The cell cannot manipulate ion concentrations for obvious reasons. That is why it uses the possibility of producing proteins preventing water crystallization [54].

Another group of proteins meeting the condition of high compatibility of the hydrophobicity distribution with the T distribution and thus reproducing the distribution present in the spherical micelle are proteins referred to as fast-folding or even ultra-fast folding [56]. The presence of water directing the process of micelle formation means that each time the denaturing agent is removed, the protein returns to its correct form only under the influence of water – in an interpretation based on the FOD model – it returns to the form of a spherical micelle.

Another protein showing high compatibility of the T and O distribution is the titin domain [57]. It is a component of muscle tissue protein which, when subjected to external stress, is able to return to its native form after the stress has subsided despite significant deformation during external force. If this return were not possible, muscle tissue would be out of control [57].

Thus, proteins with high solubility and not interacting with other molecules perform an important biological function of living organisms.

The amino acid sequence – imposed by evolution – forces neighborhood of the residues, as a result of which the reconstruction of the micelle structure is not possible. The consequence of this is the presence of a local mismatch of the O distribution to the T distribution. This mismatch is a form of recording information encoding the biological function of the protein, which biochemistry calls specificity. If the local mismatch involves the exposure of high hydrophobicity on the surface of the protein, it creates a possibility for a complex with another protein, which is also interested in eliminating adverse contact with water [49].

Similarly, the local hydrophobic deficit associated with the presence of cavity creates conditions for readiness for interaction with the ligand. The method and type of non-adaptation to idealized distribution is a form of record identified by a suitably compatible molecule, which thus interacts [48].

The record of the biological function of the protein consists in generating a specific form and degree of non-adjustment of the hydrophobicity distribution of O to the distribution of T. The amino acid sequence is a record of the inability to obtain the perfect micelle state. That is why the protein was defined as intelligent micelle [58]. Therefore, the RD parameter measures the degree of maladjustment, but also allows the identification of the place where this specific information is saved.

It is possible to determine the value of the RD parameter for a selected fragment of the chain demonstrating its role as carrying information about a potential interaction. One can also perform a stepwise elimination of residues that increase the value of RD until the value of RD becomes < 0.5. Eliminated residues allow the identification of their participation in recording information about the biological function of the protein. The part remaining after the elimination process indicates the part of the protein molecule that retains the characteristics of the micelle.

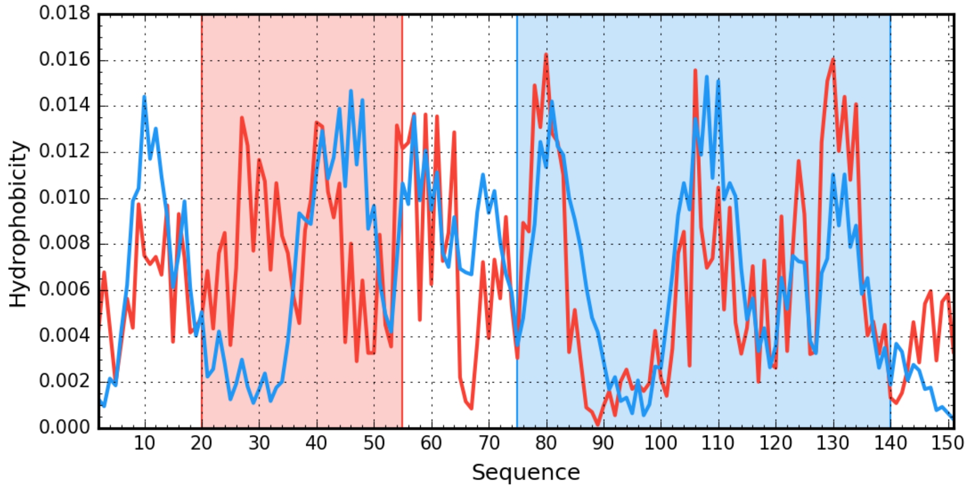

Figure BS.3. Theoretical (T – blue) and observed (O – red) hydrophobicity density profiles calculated for a chaperone protein (PDB ID: 1GME). The distinguished fragments: 20-55 (red) and 75-140 (blue) are examples of sequence fragments expressing a highly discordant and highly accordant status respectively.

A very good example is the domain A of the chaperone protein (PDB ID: 1GME [59]) with a varying degree of matching the T and O distribution. Fragment 75-140 shows a high agreement (RD = 0.321), while the discordant status of segment 20-55 is described by RD = 0.807. This fragment contains a local excess of hydrophobicity (positions 20-35) and also a local hydrophobicity deficit (positions 36-50). They both contribute to the lack of agreement between the distributions.

Based on the analysis of the hydrophobicity distribution in amyloids, they were identified as band micelles. It turns out that the chains of amyloid proteins are not able to generate the form of a globular micelle in favor of the band micelle [53].

The fuzzy oil drop model discussed here was used to describe the reasons for the diversity in the composition of the hydrophobic nucleus in proteins representing 3α versus 4β + α folds.
